# Immature Vascular Smooth Muscle Cells in Healthy Murine Arteries and Atherosclerotic Plaques: Localization and Activity

**DOI:** 10.3390/ijms23031744

**Published:** 2022-02-03

**Authors:** Alexander Balatskiy, Ilia Ozhimalov, Maria Balatskaya, Alexandra Savina, Julia Filatova, Natalia Kalinina, Vladimir Popov, Vsevolod Tkachuk

**Affiliations:** 1 Medical Scientific and Educational Centre, Lomonosov Moscow State University, 119192 Moscow, Russia; tkachuk@fbm.msu.ru; 2 Institute of Basic Neurology, Federal Center of Brain Research and Neurotechnologies, Federal Biomedical Agency, 117513 Moscow, Russia; 3 Faculty of Medicine, Lomonosov Moscow State University, 119991 Moscow, Russia; i.ozhimalov@gmail.com (I.O.); m.balatskaya@gmail.com (M.B.); alexa.savi@yandex.ru (A.S.); n_i_kalinina@mail.ru (N.K.); galiantus@gmail.com (V.P.); 4 Department of Cardiology, A.I. Yevdokimov Moscow State University of Medicine and Dentistry, Ministry of Healthcare, 127473 Moscow, Russia; filatovacardio@mail.ru; 5 Institute of Experimental Cardiology, National Medical Research Center of Cardiology, 121552 Moscow, Russia

**Keywords:** immature smooth muscle cells, pericytes, atherosclerosis, NG2, CD146, angiotensin II, angiotensin II receptor type 2

## Abstract

The local development of atherosclerotic lesions may, at least partly, be associated with the specific cellular composition of atherosclerosis-prone regions. Previously, it was demonstrated that a small population of immature vascular smooth muscle cells (VSMCs) expressing both CD146 and neuron-glial antigen 2 is postnatally sustained in atherosclerosis-prone sites. We supposed that these cells may be involved in atherogenesis and can continuously respond to angiotensin II, which is an atherogenic factor. Using immunohistochemistry, flow cytometry, wound migration assay xCELLigence system, and calcium imaging, we studied the functional activities of immature VSMCs in vitro and in vivo. According to our data, these cells do not express nestin, CD105, and the leptin receptor. They are localized in atherosclerosis-prone regions, and their number increases with age, from 5.7% to 23%. Immature VSMCs do not migrate to low shear stress areas and atherosclerotic lesions. They also do not have any unique response to angiotensin II. Thus, despite the localization of immature VSMCs and the presence of the link between their number and age, our study did not support the hypothesis that immature VSMCs are directly involved in the formation of atherosclerotic lesions. Additional lineage tracing studies can clarify the fate of these cells during atherogenesis.

## 1. Introduction

Current concepts of atherogenesis suppose that atherosclerotic plaques occur in specific sites mainly due to the shear stress, which changes when the laminar blood flow turns into turbulent curvatures and bifurcations of the arteries. Indeed, low shear stress leads to lipid accumulation and changes in expression levels of various genes in the vascular wall [1]. However, in humans, there are relatively straight sections of arteries, where atherosclerotic lesions occur often enough. Interestingly, coronary atherosclerosis does not develop in ApoE-deficient mice (a popular animal model of atherosclerosis), although the anatomy of their coronary arteries is not fundamentally different from that in humans [2].

We suppose that the local development of atherosclerotic lesions may, at least partly, be associated with the specific cellular composition of atherosclerosis-prone regions. For example, progenitor cells located in such regions could induce the development of atherosclerosis, both by differentiating into other cell types and by influencing its microenvironment by the secretion of various growth factors and microRNAs. Some candidates for such cells were discovered a long time ago. Pericyte-like stellate intimal cells were first described in 1866 by Langhaas, who considered them to be intimal fibroblasts [3]. It was demonstrated that stellate cells are abundant in atherosclerotic lesions [4]. Further studies have shown that these cells express pericytic markers [5]. It is known that pericytes accumulate lipids in experimental atherosclerosis [6]. It also was demonstrated that a population of myointimal cells (myofibroblast-like) originates from the pericytes during arterial intimal thickening formation [7]. In 2018, Roostalu et al. described a population considered as immature vascular smooth muscle cells (VSMCs) expressing pericytic markers CD146 and neuron-glial antigen 2 (NG2). It has been demonstrated that a small population of immature cells is postnatally sustained at arterial branch sites. In response to arterial injuries, these cells give rise to neointima, but on extensive damage, they are replaced by adventitial cells. Moreover, Roostalu et al. showed that these cells express yes-associated protein 1 (YAP1), which is one of the key participants in the mechanoreception signaling cascades. This suggests the sensitivity of NG2^+^CD146^+^ cells to shear stress. The authors believe that these cells are immature smooth muscle cells and not pericytes, as they respond to phenylephrine [8].

Immature VSMCs are localized exactly in the same areas where atherosclerosis occurs, but there are no up-to-date data regarding their role in atherogenesis. In the present study, we evaluated the role of these cells in atherogenesis for the first time. We estimated the influence of several factors, including hyperlipidemia, age, low shear stress, and angiotensin II.

Angiotensin II is a powerful atherogenic factor. Its infusions accelerate the development of atherosclerosis in mice [9], whereas the administration of angiotensin II type 1 (AT_1_R) receptor blockers may cause regression of early stages lesions [10]. It is known that AT_1_R activation leads to the production of reactive oxygen species in the vessel wall, in part because the AT_1_R is linked to activation of an NADH/NADPH oxidase in vascular cells [11]. This leads to the inactivation of nitric oxide and endothelial dysfunction (data reviewed in [12]). It has also been established that the expression of a receptor for oxidized LDL, known as the LOX receptor, is dramatically increased by AT_1_R-receptor activation [13]. Similar to other atherogenic factors, angiotensin II acts systemically in the entire bloodstream; however, atherosclerosis develops locally. In 2017, Sysoeva et al. demonstrated that, in most adipose-derived stem cells (which share the same origin with pericytes and VSMCs [14]), AT_1_R undergoes rapid internalization upon ligand binding. However, they described a small (about 5%) subpopulation that responds to serial applications of angiotensin II. This subpopulation co-express angiotensin II type 1 and type 2 (AT_2_R) receptors and exhibited increased adipose competency [15]. We hypothesized that if immature VSMCs in atherosclerosis-prone regions are enriched in cells continuously responding to angiotensin II, this hormone may have a more powerful effect in these sites. Therefore, we sought to find NG2^+^CD146^+^ cells that re-respond to serial applications of angiotensin II and/or co-express AT_1_R and AT_2_R.

## 2. Results

### 2.1. Immunophenotype of Cells in Bifurcations of Large Arteries

**Figure 1:**
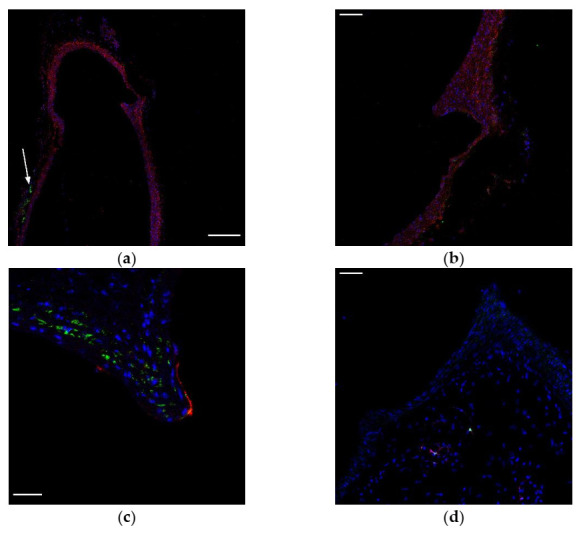
Representative images showing the bifurcation of the aorta and the innominate artery. No pericytic markers were found in atherosclerosis-prone sites (bifurcations of large arteries), except for NG2, CD146, and CD140b (which is a common marker for cells of mesenchymal origin): (**a,b**) staining with antibodies to CD140b (red) and to leptin receptor (green, arrow); (**c**) staining with antibodies to CD105 (red) and to NG2 (green); (**d**) GFP (green) and staining with antibodies to GFP (magenta) in nestin-GFP mice. In all images, nuclei are blue. Scale bars (**a**) 200 µm, (**b**,**d**) 50 µm, and (**c**) 20 µm.

To clarify whether the cells in the bifurcations of large vessels are pericytes or immature VSMCs, we stained murine aortas with antibodies to some pericytic markers: CD140b, CD105, and the leptin receptor. Platelet-derived growth factor receptor beta (CD140b) is a common marker for pericytes and VSMC [14], and it is abundant in the aortic wall, while leptin receptor was only be found in perivascular fat (Figure 1a). CD105 was found only in endothelium but not in NG2^+^ cells (Figure 1b). We also sought to find nestin-positive cells in bifurcations of large arteries. Nestin-GFP mice were used because if nestin was found in bifurcations, we could use these animals for tracing studies. Unfortunately, nestin-positive cells expressing GFP were only found in perivascular fat (Figure 1c).

### 2.2. The Number of Immature Vascular Smooth Muscle Cells Increases in Arteries of Aged Animals

**Figure 2:**
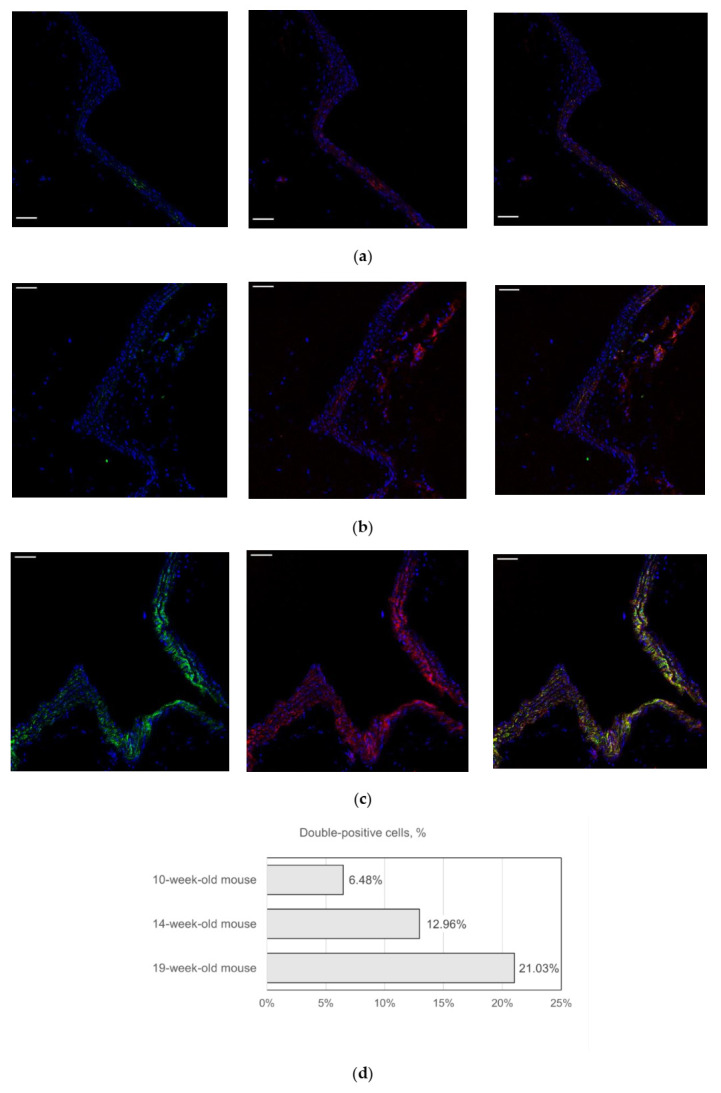
Representative images showing the bifurcations of the aortic arch and its major branches: (**a**) 10-week-old mice, (**b**) 14-week-old mice, and (**c**) 19-week-old mice. In young animals, a small number of NG2^+^CD146^+^ are located in bifurcations and other areas with low shear stress. In aged animals, the number of these cells is larger throughout all major arteries. The percentage of double-positive NG2^+^CD146^+^ cells in the vascular wall is shown on (**d**). Staining with antibodies to CD146 (red) and to NG2 (green). Nuclei are stained with DAPI (blue). Scale bars 50 µm.

During the study, we confirmed that NG2^+^CD146^+^ immature VSMCs form clusters near bifurcations of the aorta and its branches (Figure 2a,b). We also noted that the number of NG2^+^CD146^+^ immature VSMCs in the murine arteries increases significantly with age, especially after the 18th–19th week (Figure 2c).

**Figure 3:**
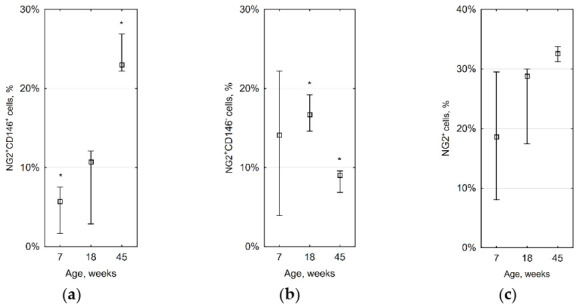
The percentage of different VSMCs changes with age: (**a**) NG2^+^CD146^+^ cells, * *p* = 0.036; (**b**) NG2^+^CD146^−^ cells, * *p* = 0.012; (**c**) all NG2^+^ cells. This means that NG2^+^CD146^−^ cells can de-differentiate into immature NG2^+^CD146^+^ cells with age. Squares show medians; whiskers show upper and lower quartiles.

To check these data, we measured the number of NG2^+^CD146^+^ cells by flow cytometry in aortas of 16 mice. We used 6 animals at the age of 6–8 weeks (oldest age used in a previous study by Roostalu et al. [8]), 5 at the age of 18–19 weeks (when we observed the increased number of immature VSMCs), and 5 at the age of 45–47 weeks (the oldest mice available in our animal facility). It was shown that their percentage in old animals increases four times (23% versus 5.7% in young ones, *p* = 0.036, Figure 3a). The percentage of NG2^+^CD146^−^ cells decreases with age (Figure 3b), while the total fraction of NG2^+^ cells does not change significantly (Figure 3c).

### 2.3. Migration and Proliferation of Immature Vascular Smooth Muscle Cells In Vitro

**Figure 4:**
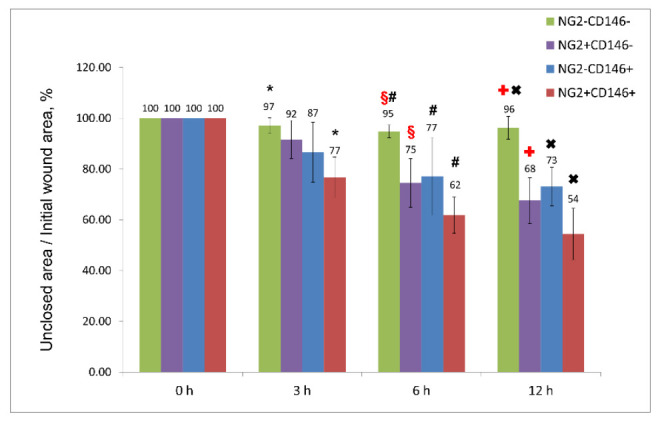
The percentage of wound closure by different subpopulations indicating the migration rate. Immature NG2^+^CD146^+^ VSMCs migrate better than other vascular cells, but the difference with mature NG2^+^CD146^−^ cells is not significant. The difference between columns marked with the same symbols is significant (*p* < 0.05).

**Figure 5:**
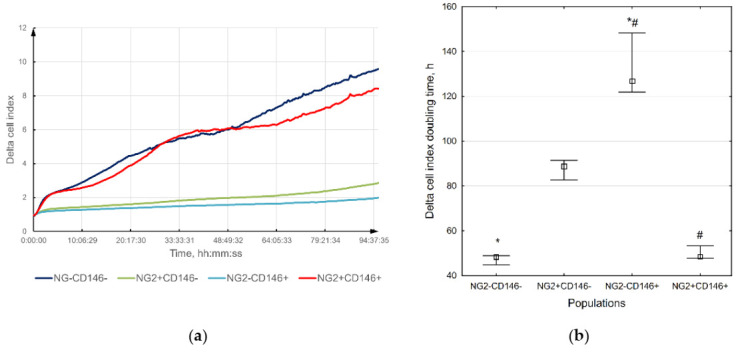
The proliferation rate of different vascular cells: (**a**) delta cell index changing over time; (**b**) delta cell index doubling time. Immature NG2^+^CD146^+^ VSMCs migrate better than other vascular cells, but the difference with mature NG2^+^CD146^−^ cells is not significant. The difference between columns marked with the same symbols is significant (*p* < 0.05). Squares show medians; whiskers show upper and lower quartiles.

The ability of immature VSMCs to migrate and proliferate was evaluated in vitro using the wound assay and xCELLigence system, respectively. It was shown that they migrate and proliferate better than other VSMCs; however, the differences with NG2^+^CD146^−^ cells were not significant (Figure 4 and Figure 5).

### 2.4. Immature Vascular Smooth Muscle Cells in Low Shear Stress Areas and in Atherosclerotic Plaques

**Figure 6:**
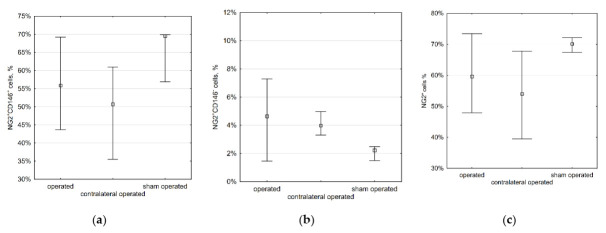
The percentage of different VSMCs cells in common carotid arteries—three months after partial carotid ligation. Immature VSMCs do not migrate into low shear stress areas and do not proliferate there: (**a**) NG2^+^CD146^+^ cells: operated, 55.9% (43.7%, 69.2%); contralateral operated, 50.7% (35.5%, 61.0%); sham operated, 69.5% (56.9%, 69.9%); (**b**) NG2^+^CD146^−^ cells: operated, 4.6% (1.5%, 7.3%); contralateral operated, 4.0% (3.3%, 5.0%); sham operated, 2.2% (1.5%, 2.5%); (**c**) all NG2^+^ cells: operated, 59.7% (47.9%, 73.4%); contralateral operated, 54.0% (39.5%, 67.8%); sham operated, 70.1% (67.4%, 72.1%). Squares show medians; whiskers show upper and lower quartiles. Data are given as median (interquartile range).

**Figure 7:**
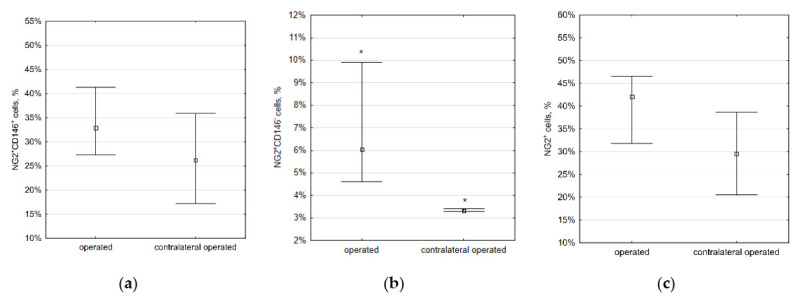
The percentage of NG2^+^ cells in common carotid arteries, two weeks after partial carotid ligation. The number of mature NG2^+^CD146^−^ (but not immature NG2^+^CD146^+^) VSMCs slightly increases in low shear stress areas: (**a**) NG2^+^CD146^+^ cells: operated, 32.9% (27.3%, 41.3%); contralateral operated, 26.2% (17.2%, 35.9%); (**b**) NG2^+^CD146^−^ cells: operated, 6.1% (4.6%, 9.9%); contralateral operated, 3.3% (3.3%, 3.4%); (**c**) all NG2^+^ cells: operated, 42.0% (31.8%, 46.5%); contralateral operated, 29.5% (20.5%, 38.7%). Squares show medians; whiskers show upper and lower quartiles. Data are given as median (interquartile range).

To evaluate the effect of low shear stress on immature VSMCs, we performed partial carotid ligation (as described in the Materials and Methods section) in six mice. Another six animals underwent a sham operation (bringing ligatures under the arteries without tying them). Three months later, the animals were euthanized, and the number of cells was assessed by flow cytometry in the operated, sham-operated, and contralateral non-operated common carotid arteries. The number of NG2^+^CD146^+^ cells in the carotid arteries was significantly higher than in the aorta, which is consistent with the literature data [8]. The percentage of NG2^+^CD146^+^ and NG2^+^CD146^−^ cells, as well as the total percentage of NG2^+^ cells, did not differ significantly in the operated and sham-operated arteries (Figure 6). Immature smooth muscle cells most likely play roles in the early stages of atherogenesis. Since in a study by Nam et al., atherosclerotic lesions in ApoE-deficient mice developed in only two weeks after partial carotid ligation, we involved another group of mice that were euthanized two weeks after surgery. Since the first experiment showed no difference between sham-operated and non-operated arteries, only six mice were included in the group, and a comparison was made between operated and contralateral arteries. In the operated arteries, the percentage of NG2^+^ cells was slightly higher (*p* > 0.05) only due to mature NG2^+^CD146^−^ (6.1% in operated arteries vs. 3.3% in contralateral arteries, *p* = 0.005) but not NG2^+^CD146^+^ VSMCs (Figure 7). Our data regarding operated and non-operated mice (see Section 2.2) show a relatively wide scatter. This can be due to large variations between individual animals, and this variation needs further study.

**Figure 8:**
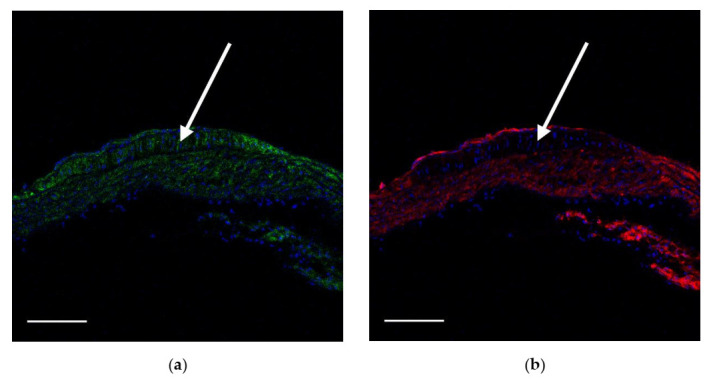

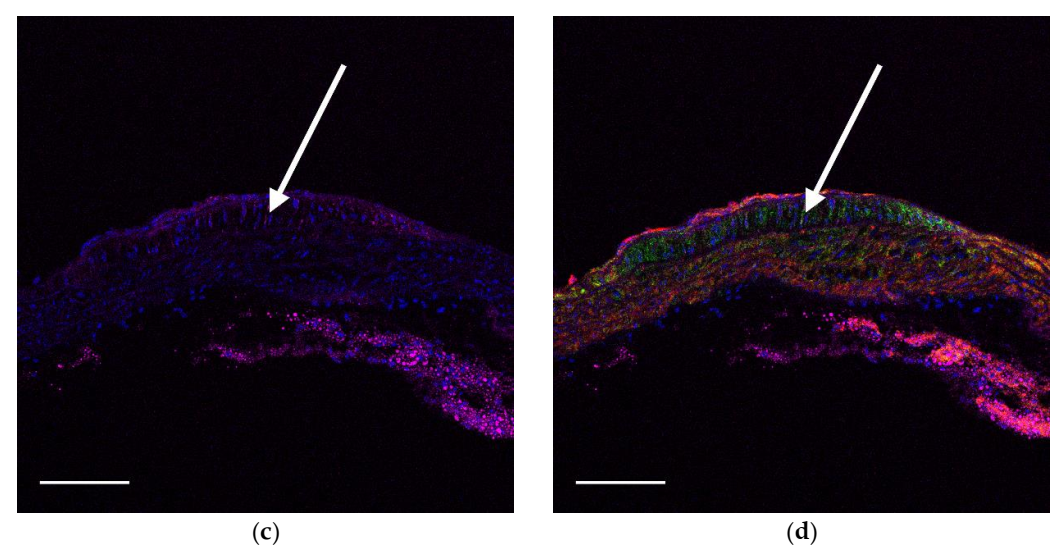
Fatty streak (arrowed) in the lesser curvature of the aortic arch in an 18-week-old ApoE knockout mouse fed with a Western-type diet for two weeks. Immature NG2^+^CD146^+^ VSMCs (green and red) are located at the streak’s borders, but inside the streak, only mature NG2^+^CD146^−^ (green only) VSMCs are present: (**a**) NG2 (green), (**b**) CD146 (red), (**c**) lipids (magenta), and (**d**) merged. Nuclei are stained with DAPI (blue). Scale bars 100 µm.

**Figure 9:**
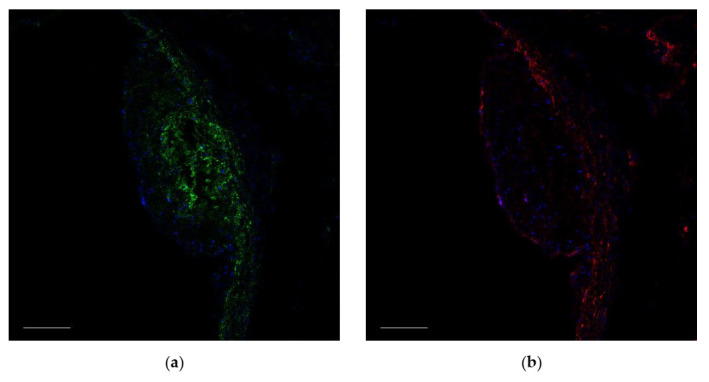

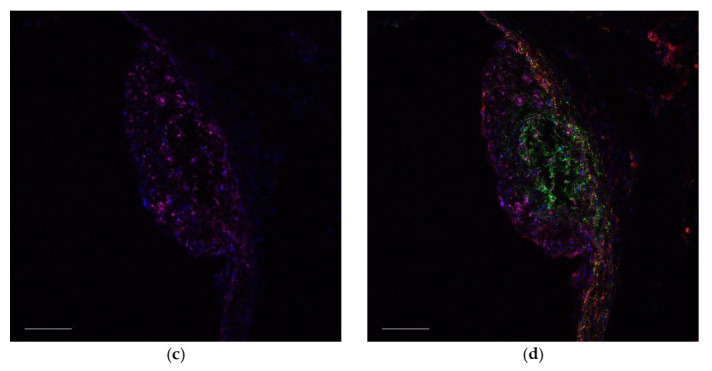
Atherosclerotic plaque in the aortic root in a 20-week-old ApoE knockout mouse fed with a Western-type diet for four weeks. Immature NG2^+^CD146^+^ VSMCs (green and red) are located at plaque’s borders, but inside the plaque, only mature NG2^+^CD146^−^ VSMCs (green only) are present: (**a**) NG2 (green), (**b**) CD146 (red), (**c**) lipids (magenta), and (**d**) merged. Nuclei are stained with DAPI (blue). Scale bars 100 µm.

**Figure A1:**
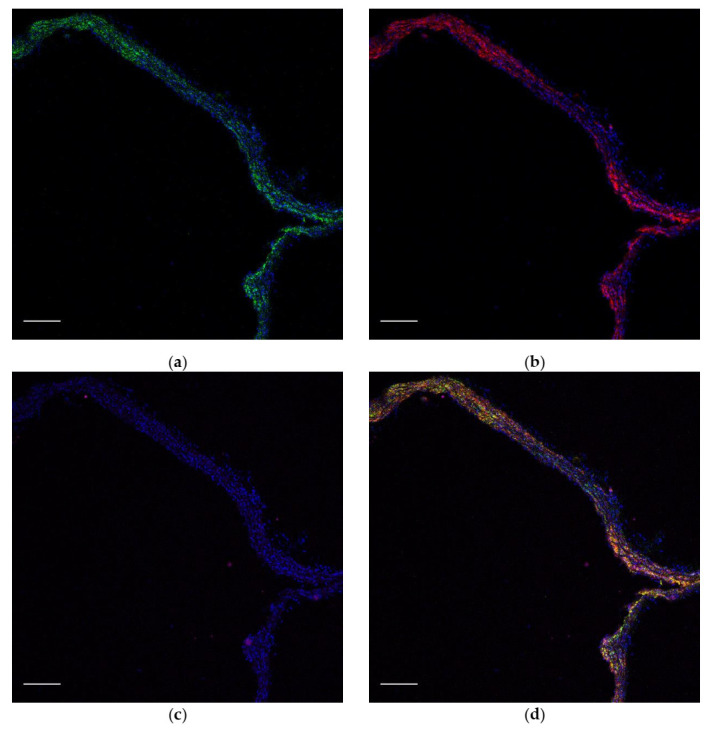
The aortic arch and the innominate artery in an ApoE knockout mouse fed with chow diet: (**a**) NG2, (**b**) CD146, (**c**) lipids, and (**d**) merged. Nuclei are stained with DAPI (blue). Scale bars 100 µm.

To check if the immature VSMCs appear in atherosclerotic plaques, we used ApoE knockout mice. In mice fed with a Western-type diet for two weeks, we observed fatty streaks where only NG2^+^CD146^−^ cells but not NG2^+^CD146^+^ cells were present (Figure 8). The same distribution was observed in advanced atherosclerotic lesions found in mice fed with a Western-type diet for four weeks (Figure 9). NG2^+^CD146^+^ were abundant in areas near atherosclerotic lesions but not inside plaques themselves. The distribution of NG2^+^CD146^+^ cells in arteries of ApoE knockout mice fed with a chow diet did not differ from the distribution in wild-type mice (Appendix A, Figure A1).

### 2.5. Angiotensin II Receptors Type 2 and the Response to Angiotensin II in Immature VSMCs

**Figure 10:**
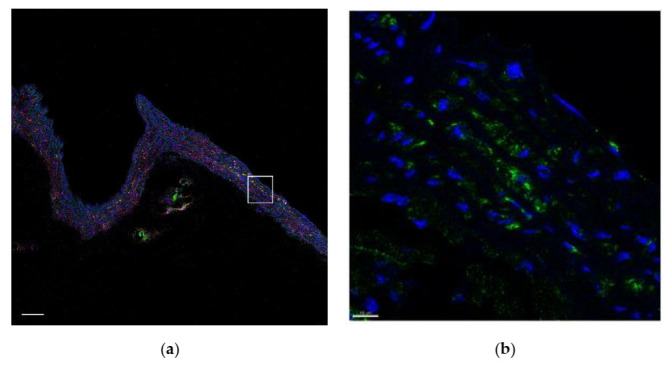

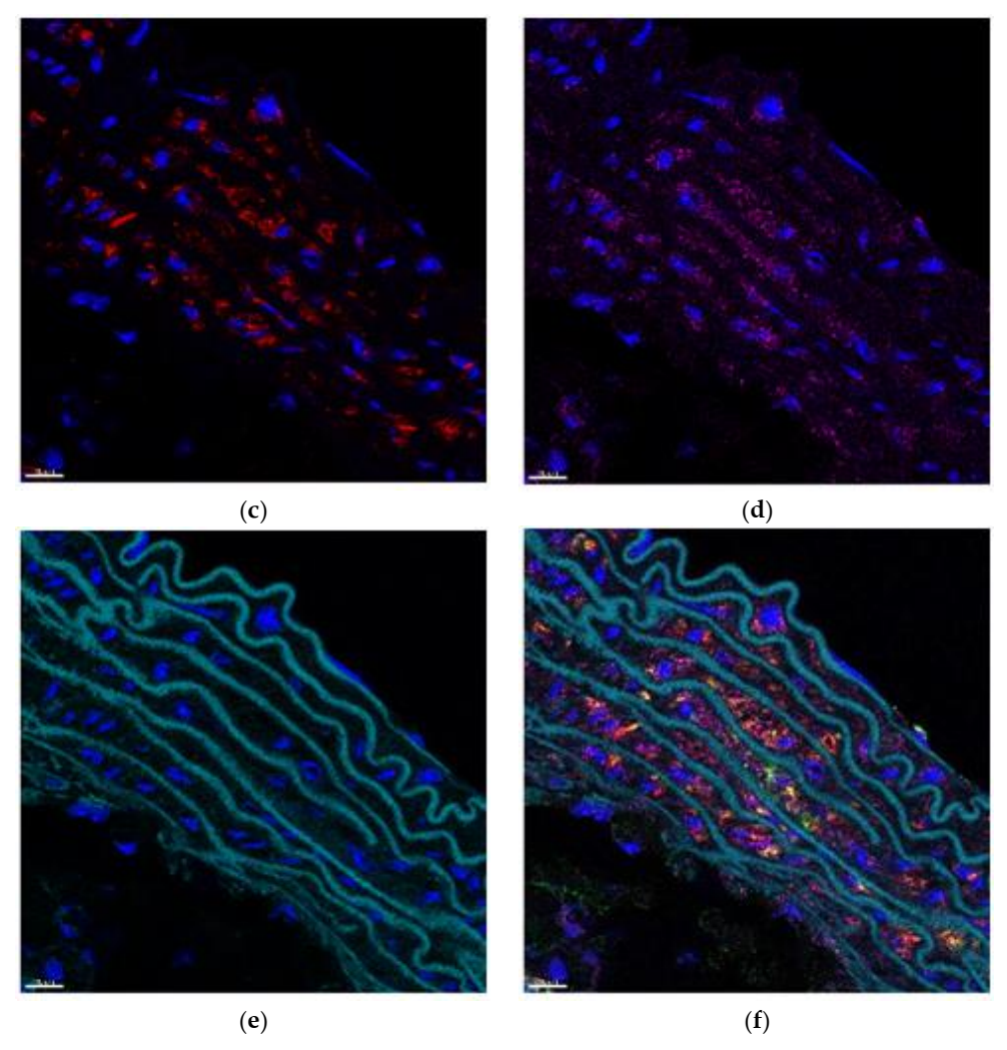
(**a**) The bifurcation of the aortic arch and the innominate artery. In such sites, immature NG2^+^CD146^+^ cells express only AT1R but not AT2R. Square indicates the area shown in (**b**–**f**)**:** VSMCs are stained with antibodies to (**b**) NG2 (green), (**c**) CD146 (red), (**d**) AT_1_R (magenta), and (**e**) AT_2_R (cyan); (**f**) merged image. On (**e**)**,** AT_2_R staining is not observed. Visible fluorescence is the autofluorescence of collagen fibers induced by a 488 nm laser. Nuclei are stained with DAPI (blue). Scalebars (**a**) 50 µm, (**b**–**f**) 10 µm.

**Figure 11:**
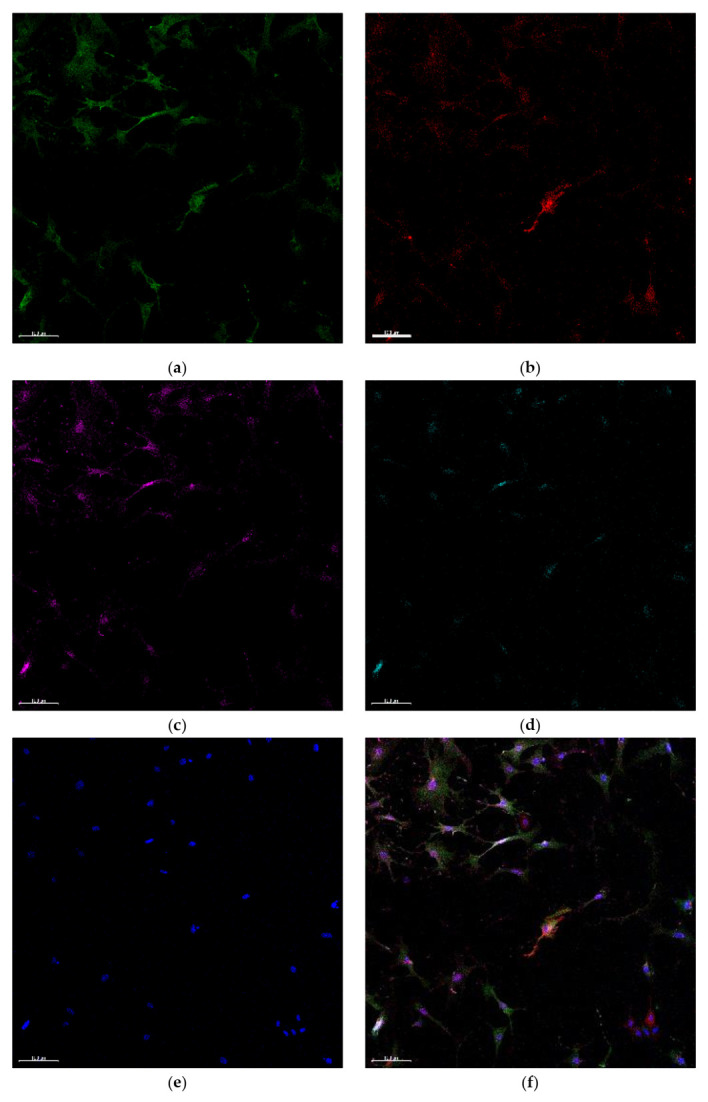
Cultured VSMCs express AT_2_R only in nuclei, and this expression is not limited to NG2^+^CD146^+^ cells: (**a**) NG2 (green), (**b**) CD146 (red), (**c**) AT_1_R (magenta), (**d**) AT_2_R (cyan), (**e**) nuclei (DAPI, blue), and (**f**) merged. Scalebars 100 µm.

**Figure A2:**
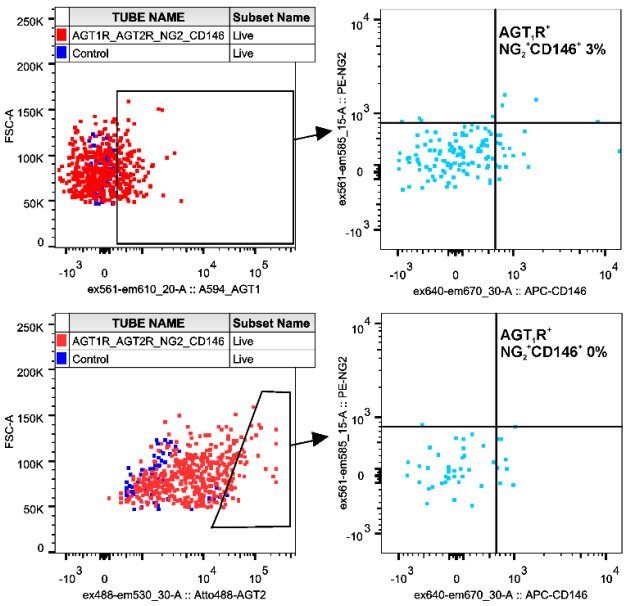
Co-expressing of NG2, CD146, AT_1_R, and AT_2_R in VSMCs. The subpopulation of AT_1_R positive cells contains immature NG2^+^CD146^+^ VSMCs, but immature VSMCs are absent in AT_2_R-positive subpopulation.

In this research, we aimed to find immature VSMCs, which can continuously respond to angiotensin II. Since such cells may express AT_2_R, we searched for those co-expressing NG2, CD146, AT_1_R, and AT_2_R. We were unable to detect cells expressing all four markers either using immunohistochemistry (Figure 10) or flow cytometry (Figure A2). NG2^+^CD146^+^ cells express only AT_1_R on the surface. In the culture of cells isolated from the aorta, AT_2_R was also not identified with standard staining (as described in the Materials and Methods section). The use of the more powerful detergent Triton-X100 revealed a weak expression of AT_2_R in the nuclei (Figure 11). Nuclear AT_1_R and AT_2_R are described in the literature. They are components of the intracellular renin-angiotensin system [16]. However, in our experiments, their expression was not strictly associated with the expression of NG2 and CD146, but these data demonstrate the performance of antibodies.

**Figure 12:**
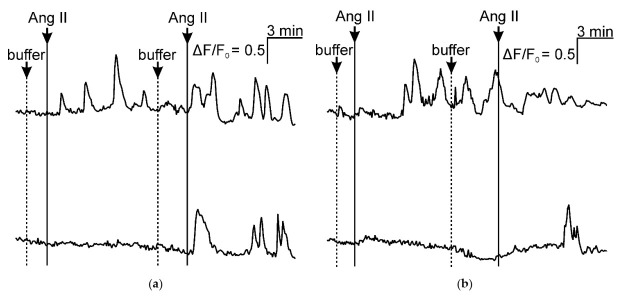
The response of sorted VSMCs to the applications of angiotensin II. Both immature and mature VSMCs have no uniform or population-specific response pattern: (**a**) NG2^+^CD146^+^ cells and (**b**) NG2^+^CD146^−^ cells.

To find VSMCs that can respond to angiotensin II continuously, we added 10^−7^ M angiotensin II to the cells loaded with calcium dye Fluo-8, registered the response for 8 min., washed cells with buffer twice, and repeated the adding of angiotensin II. In the total population isolated from murine aortae, we did not find cells responding to the re-application of angiotensin II. In sorted NG2^+^CD146^+^ immature VSMCs, we find either cells responding to both adds of angiotensin II or only to the second add (Figure 12a). The same response patterns were demonstrated for NG2^+^CD146^−^ mature VSMCs (Figure 12b).

## 3. Discussion

In the present article, we performed a first attempt to elucidate the role of immature NG2^+^CD146^+^ VSMCs in atherogenesis. These cells have very intriguing localization in sites where atherosclerosis often occurs and are supposed to be mechanosensitive [8], i.e., they may react to shear stress. Nevertheless, there are no articles demonstrating the involvement of NG2^+^CD146^+^ cells into atherogenesis directly.

There are some data regarding the effect of NG2-positive cells on the development of atherosclerosis: knockout of this gene leads to a reduction in the formation of atherosclerotic lesions and a decrease in the number of foam cells [17]. The authors of this study believe that all NG2-positive cells are synthetic smooth muscle cells. At the same time, the CD146 knockout leads to enhanced atherogenesis: after 24 weeks of a Western diet, a significant increase in atheroma in both total aortic lesion and aortic sinus of CD146-null mice was observed [18].

NG2 and CD146 are both markers of pericytes, and pericyte-like cells were previously found in the arterial wall [5], so we checked bifurcations of large arteries for expression of other pericytic markers. It is known that pericytes form matured neovessels in atherosclerotic plaques and contribute to intimal hyperplasia after a vascular injury involving the adhesion molecule ninjurin-1 [19]. Nevertheless, the processes of vascular remodeling are important in advanced atherosclerotic lesions, and little is known about the role of pericytes in the initial stages of atherogenesis. Pericytes are heterogeneous: Birbrair et al. showed that, in skeletal muscles, there are two types of pericytes: nestin^+^/NG2^+^/CD146^+^ and nestin^−^/NG2^+^/CD146^+^. Del Toro et al. demonstrated that nestin-positive cells regulate the migration of inflammatory cells during atherogenesis. In Apolipoprotein E (ApoE) knockout mice fed with a high-fat diet, nestin^+^ stromal cells from adventitia increase 30 times and contribute to the atheroma plaque [20]. The data of this study show that nestin-positive cells are located in adventitia and media but not in the intima of the arteries. Other research studies show that nestin-positive cells are located only in adventitia [21]. In nestin-GFP mice, we find a small number of nestin-positive cells in perivascular fat only. The amount of leptin receptor increases in atherosclerotic lesions [22], and many studies demonstrate lesion-promoting properties of leptin (data reviewed in [23]). In healthy murine arteries, we find leptin receptors only in perivascular fat, which indicates that leptin does not promote atherosclerosis via NG2^+^CD146^+^ cells. Our data demonstrate that, in healthy murine arteries, CD105 is expressed only by endothelium. This is consistent with what has been found in other studies [24,25]. In human arteries, it is also expressed in smooth muscle cells, and the expression increases in atherosclerotic lesions [26], so the immunophenotype of human NG2^+^CD146^+^ cells needs further investigation. Together, the present findings confirm that NG2^+^CD146^+^ cells are not pericytes.

During the study, we noted that the number of immature VSMCs in arteries markedly increases with age. This fact seems counterintuitive because Roostalu et al. demonstrated that NG2^+^CD146^+^ cells are abundant in the embryonic aorta, while in adult animals, their number is small [8]. It should be noted that in that work oldest animals were 8 weeks old. Our data demonstrate a four times increase in the number of NG2^+^CD146^+^ cells in 45-week-old mice. At the same time, the number of NG2^+^CD146^−^ cells decreases, while the percentage of all NG2^+^ cells in the aorta stays constant. This means that mature NG2^+^CD146^−^ VSMCs can de-differentiate with age. Since atherosclerosis is an age-related disease, our finding could be important indirect evidence of the immature smooth muscle cells’ participation in atherogenesis. However, we did not demonstrate any increase in the number of NG2^+^CD146^+^ cells in low shear stress areas created by partial carotid ligation. These cells also do not have a significantly increased ability to migrate and proliferate. In low shear stress areas created in ApoE knockout mice, atherosclerosis develops rapidly [27], so if immature smooth muscle cells play any role in atherogenesis, they should appear there. It should be noticed that the percentage of immature VSMCs is highly variable between animals; therefore, the capability to compare groups is limited. According to our data, NG2^+^CD146^+^ cells also do not appear in atherosclerotic lesions (both fatty streaks and advanced plaques), but they are present in borders of lesions. Since NG2^+^CD146^+^ cells pre-exist in atherosclerosis-prone areas, they can stay there in a quiescent state or can proliferate and affect lesion formation. Future lineage tracing studies can clarify if NG2^+^CD146^−^ cells inside lesions arise from NG2^+^CD146^+^ cells located in the vascular wall and whether the number of NG2^+^CD146^+^ cells increases in borders of lesions.

Based on data from Sysoeva et al. [15], we sought to find cells that continuously respond to applications of angiotensin II and/or co-express AT_1_R and AT_2_R among NG2^+^CD146^+^ cells. If we discovered such cells, located in atherosclerosis-prone areas, it would be possible to investigate why angiotensin II affects atherogenesis locally. Unfortunately, the expression of AT_2_R was found neither in bifurcations of arteries nor in cultured VSMCs. Some immature VSMCs re-responded to the second addition of angiotensin II, but their response was neither unique nor uniform throughout the population. The same patterns were observed in mature NG2^+^CD146^−^ cells, so even though some VSMCs can re-respond to serial applications of angiotensin II, it is not an exclusive feature of NG2^+^CD146^+^ immature cells. It is possible to conclude that cells continuously responding to angiotensin II seems not to be involved in the local atherogenic effects of this hormone.

Thus, our study did not provide direct evidence that immature VSMCs are directly involved in atherogenesis. However, our research has several limitations. First, rodents do not form atherosclerosis spontaneously, and the cellular composition of their arteries may differ from human ones significantly. Second, we only used antibodies but not lineage tracing. For this reason, we cannot determine the origin of immature or mature VSMCs observed in situ. Additional studies can clarify the fate of immature VSMCs during atherogenesis.

## 4. Materials and Methods

### 4.1. Animals

C57Bl/6N mice were obtained from Laboratory Animal Breeding Centre “Puschino” (Puschino, Russia). Nestin-GFP mice [28] were obtained from Grigory Enicolopov from Cold Spring Harbor Laboratory. ApoE knockout mice were purchased from Jackson Laboratory (Bar Harbor, ME, USA, Stock #002052 B6.129P2-Apoetm1Unc/J). Animal housing and research procedures were conducted in compliance with Directive 2010/63/EU and approved by MSU Bioethical Committee.

### 4.2. Excision of the Aorta and Primary Culture of Mouse Aortic Wall Cells

To obtain preparations of the aorta, the animals were euthanized with carbon dioxide. The left ventricle and aorta were perfused with 5 mL of phosphate-buffered saline (PBS) to remove blood. If aorta was used for cryosectioning, it was additionally perfused with 5 mL of 4% paraformaldehyde (PFA, Sigma-Aldrich, Burlington, MA, USA) in PBS. Then, the aorta was isolated from the heart up to the renal arteries. Perivascular fat was carefully removed using a stereomicroscope. For cryosectioning, aortas were flash frozen in Tissue-Tek OCT Compound (Sakura Finetek, Torrance, CA, USA). For isolation of cells, the aortas were cut into small fragments and incubated in Dulbecco’s modified Eagle’s medium (DMEM, Gibco, Waltham, MA, USA), supplemented with 5% fetal bovine serum (FBS, Gibco, Waltham, MA, USA), 20 mM HEPES, 1 μg/mL ciprofloxacin, and 1.5 mg/mL collagenase I (Worthington, Lakewood, NJ, USA) overnight. The resulting cell suspension was passed through a 40 μm cell strainer and was directly used for flow cytometry or seeded on 24-well plates coated with rat tail collagen I (Gibco, 5 μg/cm^2^ according to the manufacturer’s protocol). Cells were cultured in DMEM supplemented with 15% FBS and 1x penicillin–streptomycin solution (Gibco, Waltham, MA, USA).

### 4.3. The Model of Low Shear Stress Conditions In Vivo

A partial carotid ligation model described by Nam et al. [27] was used to create low shear stress conditions in murine carotid arteries. Surgery was performed in C57BL/6 mice under inhalation anesthesia with isoflurane. Neck hair was removed with depilatory cream, and the skin was treated with 70% ethanol. The 5–6 mm skin incision was made along the neck, the thyroid gland was retracted, and the left external and internal carotid arteries were isolated. The internal carotid artery, together with the occipital artery, as well as the external carotid artery above the superior thyroid artery, were ligated with 7-0 prolene. In control animals, ligatures were placed under the vessels and left without tying. The skin was sutured with 5-0 silk. After euthanasia, the isolation of the carotid arteries was performed similarly to the isolation of the aorta.

### 4.4. Immunohistochemistry

The slides with 10 μm cryosections were fixed in a 4% PFA for 10 min, washed in PBS with 0.3% Tween 20 (Sigma-Aldrich, Burlington, MA, USA), and blocked in PBS containing 10% donkey serum, 1% bovine serum albumin, and 0.12% Tween 20. The slides were incubated with primary antibodies overnight at +4 °C. After incubation, the slides were washed with PBS, with 0.3% Tween 20, and incubated with secondary antibodies in PBS, with 1% bovine serum albumin, and 0.15% Tween 20 for 1 h, at room temperature, in the dark. When the visualization of lipids in atherosclerotic plaques was needed, slides were incubated with 20 µM BDP 493/503 lipid stain (12310 Lumiprobe, Moscow, Russia) for 30 min, at room temperature, in the dark. Then, the slides were incubated in PBS containing 2 μg/mL DAPI (Sigma, Burlington, MA, USA) for 10 min, washed with PBS, and coverslipped using Aqua-Poly/Mount medium (Polysciences, Warrington, PA, USA). A Zeiss LSM 780 confocal microscope was used for visualization.

### 4.5. Flow Cytometry and Cell Sorting

**Figure A3:**
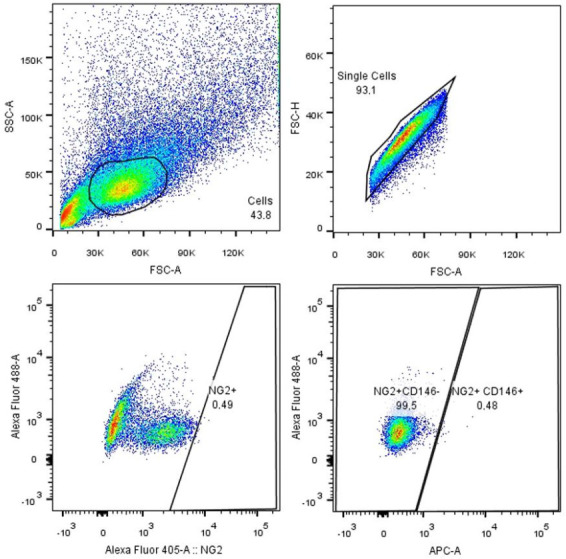
The gating strategy for calculating the percentage of NG2^+^CD146^+^ cells and for cell sorting.

For flow cytometry, isolated cells were used without cultivation. For sorting, cultured cells at passage 6 were used. Cells were detached from dishes with HyQTase Cell Detachment Reagent (HyClone, GE Healthcare Life Sciences, Piscataway, NJ, USA) and washed with Dulbecco′s phosphate-buffered saline (DPBS), with 1% BSA. All operations were performed at room temperature. Cells were blocked in DPBS with 10% FBS for 30 min and then incubated with primary antibodies in DPBS with 1% BSA for 1 h, washed with DPBS with 1% BSA, incubated with secondary antibodies in DPBS with 1% BSA for 30 min, washed DPBS with 1% BSA, and resuspended in 200 μL (600 μL for sorting) DPBS with 1% BSA. For flow cytometry, the FACS Canto II flow cytometer (BD Biosciences, Franklin Lakes, New Jersey, USA) was used. Analysis was performed using the FlowJo software. For cell sorting, FACSAria III (BD Biosciences) was used. The gating strategy is shown in Figure A3.

### 4.6. Antibodies

The following primary antibodies were used: antibodies to the leptin receptor (ab5593, 1:100, Abcam, Cambridge, UK), to CD140b (PDGFRβ) (14-1402-82, 2 μg/mL, Thermo, Waltham, MA, USA), to NG2 (AB5320, AB5320B for biotinylated, 1:100 for immunohistochemistry, 1:50 for flow cytometry, Millipore, Burlington, Massachusetts), to CD105 (MAB1320, 1:50, R&D Systems, Minneapolis, MI, USA), to (CGY)FP (AB121, 1:1000, Evrogen, Moscow, Russia), to angiotensin II receptor type 1 (AT_1_R) (SAB2500038, 10 μg/mL, Sigma-Aldrich), to angiotensin II receptor type 2 (AAR-012-AG, 1:50, Alomone Labs, Jerusalem, Israel), to CD146 (134701, 2 μg/mL for immunohistochemistry 0.5 μg/mL for flow cytometry, Biolegend, San Diego, CA, USA).

The following secondary antibodies were used: Alexa Fluor 647 AffiniPure Donkey Anti-Rabbit IgG (H+L) (711-605-152, 4.8 μg/mL, Jackson Immunoresearch, West Grove, Pennsylvania, USA), Alexa Fluor 594 Donkey anti-Rat IgG (H+L) (A-21209, 2 μg/mL, Invitrogen, Waltham, Massachusetts, USA), APC AffiniPure F(ab’)₂Fragment Donkey Anti-Rat IgG (H + L) (Jackson Immunoresearch 712-136-153, 5 μg/mL), Alexa Fluor 594 Donkey anti-Goat IgG (H + L) (Invitrogen A-11058, 4 μg/mL for flow cytometry, 8 μg/mL otherwise), BV421 Goat Anti-Rabbit IgG (BD Biosciences, 565014, 2 μg/mL), DyLight405 AffiniPure Donkey Anti-Rabbit IgG (H+L) (Jackson Immunoresearch 711-475-152 1:100), and R-Phycoerythrin Streptavidin (Jackson Immunoresearch 016-110-084, 1: 150).

Rabbit (sc-2027, 8 μg/mL, Santa Cruz Biotechnology, Dallas, TX, USA) and rat (559073, 2 μg/mL, BD Pharmingen, San Diego, CA, USA) immunoglobulins were used as negative controls. Antibodies to AT_2_R were incubated with the blocking peptide supplied with the antibodies in a 1:1 ratio for 1 h and then used as a negative control.

### 4.7. Detection of Intracellular Calcium

The cells were plated in 24-well plates one day prior to measurement. Before measurement, cells were washed with Hanks’ solution with 20 mM HEPES and incubated with a Fluo-8 fluorescent calcium probe (4 μM, Abcam) for 1 h, in the dark. The angiotensin II (Sigma-Aldrich, Burlington, MA, USA) at final concentration 10^−7^ M was added to cells twice. Between adds, cells were washed using Hanks’ solution with 20 mM HEPES. Ionomycin (Abcam, Cambridge, UK) at final concentration 10^−5^ M was used as a positive control.

### 4.8. Evaluation of Cell Migration and Proliferation

To evaluate the migration activity of the cells, the wound healing assay was used. Cells were seeded in double-well inserts (80209, ibidi, Gräfelfing, Germany) and cultured until confluence was achieved. Thereafter, the inserts were removed, creating a 500 µm wound. The images were taken every 10 min for 12 h using a Nikon Ti microscope and phase contrast. The percentage of wound closure was calculated using the ImageJ software.

To assess cell proliferation, the xCELLigence system (Agilent, Santa Clara, CA, USA) was used. This system measures the electrical resistance of cells with subsequent calculations of the Cell Index and Delta Cell Index, which are directly proportional to the number of cells.

### 4.9. Statistical Analysis

Continuous variables were expressed as median (interquartile range) and analyzed with the Mann–Whitney U test. For multiple comparisons, the Kruskal–Wallis test was used. In all figures, squares show medians; whiskers show upper and lower quartiles.

## 5. Conclusions

**Figure 13:**
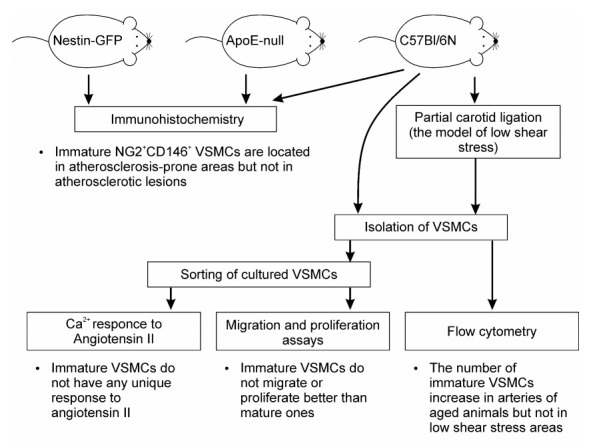
The showing all experiments and findings.

Previously, it was demonstrated that NG2^+^CD146^+^ immature VSMCs are localized exactly in the same areas where atherosclerosis occurs. In the present article, we confirmed this finding and demonstrated that the number of such cells is increased in arteries of aged mice. Other data from our research did not support the hypothesis that immature VSMCs are directly involved in the formation of atherosclerotic lesions. The diagram demonstrating all experiments and brief conclusions is present in Figure 13. Additional lineage tracing studies can clarify the role of these cells in atherogenesis. Characterization of proteins and extracellular vesicles secreted by immature VSMCs can also be helpful.

## Data Availability

The data presented in this study are available on request from the corresponding author.
